# Increased Placental Glucose Transport Rates in Pregnant Mice Carrying Fetuses with Targeted Disruption of Their Placental-Specific *Igf2* Transcripts Are Not Associated with Raised Circulating Glucose Concentrations

**DOI:** 10.1155/2011/171376

**Published:** 2011-02-08

**Authors:** Clive J. Petry, Mark L. Evans, Dianne L. Wingate, Ken K. Ong, Wolf Reik, Miguel Constância, David B. Dunger

**Affiliations:** ^1^Department of Paediatrics, University of Cambridge, Box 116, Addenbrooke's Hospital, Hills Road, Cambridge CB2 0QQ, UK; ^2^Department of Medicine, University of Cambridge, Cambridge CB2 0QQ, UK; ^3^Institute of Metabolic Science, University of Cambridge, Cambridge CB2 0QQ, UK; ^4^M.R.C. Epidemiology Unit, Addenbrooke's Hospital, Cambridge CB2 0QQ, UK; ^5^Babraham Institute, Babraham CB22 3AT, UK; ^6^Centre for Trophoblast Research, University of Cambridge, Cambridge CB2 3EG, UK; ^7^Department of Obstetrics & Gynaecology, University of Cambridge, Cambridge CB2 0SW, UK

## Abstract

At the beginning of the third week of pregnancy, mouse fetuses with targeted disruption of their paternally-transmitted insulin-like growth factor 2 gene placental-specific transcripts have growth-restricted placentas but normal body weights due to upregulated placental nutrient transport. We assessed whether increased placental glucose transport rates were associated with raised maternal glucose concentrations by performing intraperitoneal glucose tolerance tests (ipGTT) in pregnant mice carrying knockout pups and comparing them with mice carrying genotype-matched phenotypically wild type pups. Mean ± SD body weights of affected pups were 95 ± 8% of control values at e16 and 73 ± 7% at e18. There were no differences in areas under the maternal ipGTT curves at either e16 (mean ± SD being 99.0 ± 9.1% of control values; *P* = .9) or e18 (91.4 ± 13.4%; *P* = .3), suggesting that effects on transplacental glucose transport in these mice are not mediated through changes in maternal glucose concentrations.

## 1. Introduction

The murine insulin-like growth factor 2 gene (*Igf2*) is imprinted such that in pregnancy only the paternally inherited copy of the gene is expressed in both the fetus and placenta where the protein that it codes for, Igf-ii, acts as a key fetal growth factor [1]. Mouse fetuses with disrupted *Igf2 *transcripts expressed exclusively by the labyrinthine trophoblast of the placenta (P0-*Igf2*) are growth restricted in late pregnancy such that at birth they weigh on average 31% less than their wild type litter mates [2]. In contrast to mice with the full *Igf2 *gene disrupted where placental and fetal growth restrictions occur concurrently [3], the fetal growth restriction in P0-*Igf2 *null mice is preceded by placental growth restriction such that on day 14 of pregnancy, whilst there is no effect on fetal weights, placental weights of affected fetuses weigh on average 18% less than those of unaffected fetuses. By day 16, however, whilst placentas of affected fetuses weigh on average 32% less than those of unaffected fetuses, the first signs of fetal growth restriction become evident with affected fetuses weighing on average 4% less than unaffected litter mates. Just prior to parturition, weights on day 19 of pregnancy are reduced by 32% for the placenta and 22% for the fetus [2]. The above results suggest that around day 14 of pregnancy, the placenta is somehow able to compensate for its own growth restriction by upregulating its nutrient supply to the fetus such that body weights are maintained [4]. By day 16, this compensation helps limit the fetal growth restriction to a large extent despite the placenta being around a third underweight, whereas later in the pregnancy, the placenta appears to become overwhelmed and the fetal weight restriction approaches that of the placenta.

The compensatory increase in nutrient supply relative to placental weight that occurs around the start of the third week of the mouse pregnancy in this model includes a greater than 50% increase in placental glucose transfer rates on day 16 of pregnancy when they are expressed relative to placental weights [4, 5]. This increase appears to be at least partly mediated by increased placental *Slca3 *(glut3) expression. The fact that glucose transfer is normalised at this stage of pregnancy despite having a severely growth restricted placenta is emphasised by the fact that the placental glucose transfer rates in affected fetuses are similar to those of unaffected litter mates when expressed relative to fetal weights. By day 19, when both the fetus and the placenta are severely growth restricted there is still increased placental glucose transport but it is of a smaller magnitude of around 30% above that of control values [5]. *Igf2*, at least in the first half of the third week of pregnancy in the mouse, therefore appears to be able to upregulate maternal nutrient supply to meet fetal demand [6]. We have recently suggested that such fetal demand could be affected by fetal genotype effects on maternal metabolism [7]. We therefore performed the following study to test the hypotheses that the relative maintenance of fetal weight and the increased placental glucose transport at day 16 of pregnancy in this mouse model is related to increased maternal glucose concentrations and that fetal growth restriction later in pregnancy is related to a failure of the placenta to influence maternal metabolism and a subsequent lowering of maternal glucose concentrations.

## 2. Materials and Methods

### 2.1. Animals

All experiments were performed under the Animals (Scientific Procedures) Act 1986 and were approved by the University of Cambridge Animal Ethics Committee. The mice were kept under controlled conditions with a 12 h light/dark cycle. They had free access to food and water throughout (except for the starvation period immediately prior to the glucose tolerance test when water was still freely available).

### 2.2. Genotyping and Experimental Groups

The P0-*Igf2^+/−^* mice, where originally a 5 kb BamHI genomic fragment was replaced by a loxP site in the disrupted P0 allele [2], were bred on C57Bl/6 backgrounds. The genetic status of the offspring was tested by PCR reactions using ear biopsy genomic DNA extracted as per the manufacturer's instructions (using Qiagen DNeasy Blood and Tissue kits; Qiagen, Crawley, West Sussex, U.K.) as previously described [2, 5].

**Figure 1:**
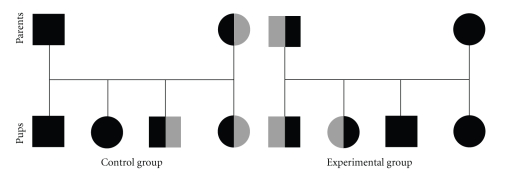
Schematic of the matings in the P0-*Igf2* knockout study. Male mice are represented by squares and female mice by circles. Wild-type mice are represented by solid black shapes and heterozygote knockout mice by shapes that are half solid black and half grey (maternal inheritance of the disrupted allele is represented by the grey half being on the right hand side, and paternal inheritance with the grey half being on the left hand side). Penetrance of the knockout gene is estimated to be 50% in each case, and half of the offspring are assumed to be males. In each case the control mothers, whilst they were heterozygous P0-*Igf2 *knockouts, were phenotypically wild type due to having inherited their disrupted allele from their mothers and imprinting.

Experimental females were wild-type C57Bl/6 mice (purchased from Charles River Ltd., Margate, Kent, U.K.) who were mated with P0-*Igf2 *heterozygous knockout male mice (Figure 1). Control females were heterozygous P0-*Igf2^−/+^* knockouts who were phenotypically wild-type due to having inherited disrupted and *imprinted *P0-*Igf2 *from their mothers, who subsequently were mated to wild-type C57Bl/6 males (Figure 1). In both groups the pregnant females would therefore have been carrying litters where approximately half the fetuses were wild type and half were genotypically heterozygous knockouts. Due to imprinting, the genotype but not the gene expression distribution amongst the pups was therefore the same for the two groups. The difference between them was whether the disrupted gene was usually expressed or whether it was usually imprinted such that disrupting it had no consequence. Pregnancy was assumed at the expulsion of a vaginal plug, although for the studies on days 16 and 18 of pregnancy, only those animals who gained weight and/or had palpable pups suggestive of them being pregnant were assessed. The day that the plug was found was considered to be day 0 (e0) of pregnancy.

### 2.3. Glucose Tolerance Tests

5–10 mice from each group had their glucose tolerance test (GTT) assessed on either day 1, 16, or 18 of pregnancy by injecting them intra-peritoneally with 1 g/kg body weight glucose (administered as a 10% (w/v) solution) after a 15-hour fast as previously described [8]. Blood glucose measurements were taken 0, 15, 30, 60, 120, and 180 minutes after the glucose injection.

### 2.4. Blood Glucose and Serum Insulin Concentrations

Blood glucose measurements were made using a Hemocue 201+ glucose meter (Hemocue Ltd., Sheffield, U.K.). Fasting and 180 minutes post load serum insulin concentrations were measured by ELISA (Rat and Mouse Insulin ELISA, Millipore, London, U.K.) according to the manufacturer's instructions.

### 2.5. Indirect Indices of Insulin Sensitivity

Fasting insulin sensitivity was assessed indirectly using fasting insulin and glucose concentrations and the HOMA calculator ([9]; available from http://www.dtu.ox.ac.uk/index.php?maindoc=/homa/index.php). Whilst the underlying principles of HOMA modelling are invalid in rodents [10], the values produced by this model do correlate significantly, albeit modestly in some studies, with those measures of insulin sensitivity gained from hyperinsulinaemic, euglycaemic clamps in both mice [11] and rats [12, 13] without the need for prior surgery or general anaesthesia. The insulin/glucose ratio was used as an indirect index of insulin sensitivity (at 180 minutes after the glucose injection), having previously been used as such in rodents [12, 14].

### 2.6. Statistical Analysis

Maternal blood glucose concentrations were assessed at both individual time points and as an integrated area under the full GTT curve calculated using the trapezoid rule. Based on performing GTTs on at least 14 mice in total per day of pregnancy and GTT results from control mice from our previous study [8], this study had 80% statistical power to detect a significant difference (*α* = 0.05) in areas under the glucose curve of 174.6 mmol·min/L (equivalent to a rise of approximately 1.0 mmol/L across the GTT). When comparing data from two groups on a particular day of pregnancy, the Mann Whitney *U* test was used. Where overall comparisons were made using all 3 days of pregnancy in the same model, two-way ANOVA was used with the experimental group and day of pregnancy as fixed variables and area under the GTT curve as the dependent variable. All statistical analysis was performed using SPSS for Windows, version 14.0 (SPSS Inc., Chicago, USA). Data are presented as mean ± standard deviation unless stated otherwise, showing results from the experimental group followed by those from the control group. *P* < .05 was considered statistically significant throughout.

## 3. Results

### 3.1. Day 1 of Pregnancy

On day 1 of pregnancy, there was no detectable difference in any of the GTT glucose concentrations or in area under the GTT curves between experimental and control groups (Table 1). There was no detectable difference in fasting insulin sensitivity (HOMA %S; values being median (interquartile range); *n* = 10 per group): 141.8 (115.9~146.7) versus 140.7 (61.2~147.4), respectively (*P* = .9). Neither was there a difference in insulin/glucose ratio 180 minutes after the glucose injection, median (interquartile range) values being 6.4 (5.8~113.9) (*n* = 10) versus 13.9 (5.9~70.9) (*n* = 10) (all × 10^−3^) (*P* = .5).

### 3.2. Day 16 of Pregnancy

**Figure 2:**
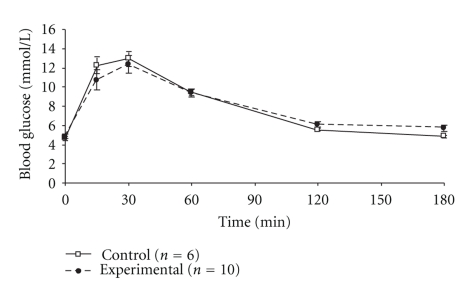
Intra-peritoneal glucose tolerance tests of pregnant mice carrying litters containing P0-*Igf2^+/−^* knockout pups on day 16 of pregnancy. *P < .05.

On day 16 of pregnancy P0-*Igf2^+/−^* pups weighed 0.62 ± 0.05 g (*n* = 36) and wild-type (P0-*Igf2^+/+^*) pups weighed 0.65 ± 0.06 g (*n* = 81) (*P* = .001). There was no detectable difference in area under the maternal GTT curves: 1428 ± 189 (*n* = 10) versus 1414 ± 130 (*n* = 6) mmol·min/L (*P* = 1.0, Figure 2) or in blood glucose concentrations between 0~120 minutes after the glucose injection (all *P* > .05; Figure 2). However, experimental mice did have higher blood glucose concentrations than controls 180 minutes after the glucose injection: 5.7 ± 0.9 (*n* = 10) versus 4.8 ± 0.6 (*n* = 6) mmol/L (*P* = .04). There were no detectable differences in fasting insulin sensitivity between mice carrying P0-*Igf2^+/−^* offspring and controls (HOMA %S; values being median (interquartile range)): 107 (15~154) (*n* = 10) versus 152 (115~156) (*n* = 6) (*P* = .3). Neither was there a difference in insulin/glucose ratio 180 minutes after the glucose injection, median (interquartile range) values being 10.0 (6.1~24.0) (*n* = 10) versus 8.0 (6.5~14.1) (*n* = 6) (all × 10^−3^) (*P* = .8).

### 3.3. Day 18 of Pregnancy

**Figure 3:**
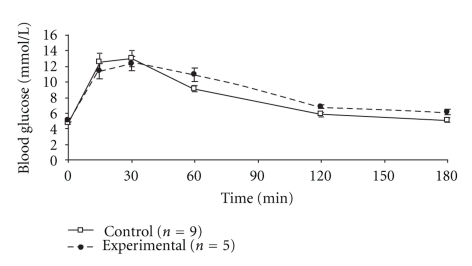
Intra-peritoneal glucose tolerance tests of pregnant mice carrying litters containing P0-*Igf2^+/−^* knockout pups on day 18 of pregnancy.

On day 18 of pregnancy P0-*Igf2^+/−^* pups weighed 0.97 ± 0.1 g (*n* = 21) and wild-type (P0-*Igf2^+/+^*) pups weighed 1.33 ± 0.11 g (*n* = 86) (*P* < .0001). There was still no detectable difference in area under the maternal GTT curves between the P0-*Igf2* experimental mice and controls: 1566 ± 229 (*n* = 5) versus 1431 ± 209 (*n* = 9) mmol·min/L (*P* = .6, Figure 3). Neither were there significant differences between the groups at any of the individual time points although there were trends for higher glucose concentrations in the experimental mice 120 minutes and 180 minutes after the glucose injection: 6.8 ± 0.8 (*n* = 5) versus 5.8 ± 0.9 (*n* = 9) (120 minutes; *P* = .1) and 6.1 ± 1.1 (*n* = 5) versus 5.1 ± 0.8 (*n* = 9) (180 minutes; *P* = .05). There were no differences in fasting insulin sensitivity between mice carrying functionally knockout pups and controls (HOMA %*S*; values being median (interquartile range)): 11 (4~147) (*n* = 5) versus 54 (23~153) (*n* = 9) (*P* = .1). Neither was there a difference in insulin/glucose ratio 180 minutes after the glucose injection, median (interquartile range) values being: 10.7 (5.1~28.1) (*n* = 5) versus 12.9 (6.6~39.5) (*n* = 9) (all × 10^−3^) (*P* = .5).

## 4. Discussion

Results from this study suggest that the increased placental glucose transport and relative maintenance of fetal weight on day 16 of pregnancy in mice with targeted disruption of their paternally-inherited P0-*Igf2 *transcript [2, 5] are not related to higher circulating maternal glucose concentrations. There was good reason to suspect that early in the third week of pregnancy (e.g., day 16 around the time when a growth spurt should begin) the P0-*Igf2 *knockout model fetal nutrient demand would have been met through fetal genotype effects on maternal metabolism as blood glucose concentrations are raised in the affected pups (Constância, unpublished observations) and the observed increase in placental glucose transport rates [5] could only occur with a sufficient glucose supply. The lack of evident alterations in maternal glucose concentrations in the present study would suggest that the difference in total blood volumes between the pregnant mother and her pups is sufficiently large that the extra glucose crossing the placenta and leaving the maternal blood is such a small fraction of the total amount of glucose within the pool that it can be achieved without the need for more major changes in maternal glucose concentrations.

We recently established, following indirect evidence for this in human studies (reviewed in [15]), that the fetal genotype can have an effect on maternal glucose concentrations when phenotypically wild-type mice were shown to have raised glucose concentrations (particularly) on day 16 of pregnancy when they were carrying pups with a maternally inherited 13 kb region of DNA that disrupted the whole *H19 *gene and the nearby *Igf2 *control element [8]. This model has increased *Igf2 *expression and placental and fetal weights [16, 17]. In contrast, it seems that disrupting P0-*Igf2 *transcript in mice rather than enhancing it removes any evidence of a fetal genotype effect on maternal glucose concentrations. Preliminary results from experiments where wild-type pregnant mice carried pups all of which had a disrupted paternally transmitted P0-*Igf2 *transcript (resulting from the mating of a wild-type female mouse with a male that is homozygous for the disrupted P0-*Igf2* transcript) also failed to show an alteration in maternal glucose concentrations (Sferruzzi-Perri and Fowden, personal communication). This suggests that the lack of an apparent fetal genotype effect on maternal glucose concentrations in our model is not due to the weakness of a fetal-genotype-mediated placental signal to the mother to increase glucose availability which might have been caused by only half of the pups being affected [7]. Instead, especially if the signal is related to *Igf2 *expression, disrupting P0-*Igf2 *might remove all or a large fraction of the signal that alters maternal glucose concentrations. Whilst it did not cause raised maternal glucose concentrations, neither did it cause a lowering of maternal glucose concentrations in this model, although that could result from the fact that the P0 transcript makes up only a fraction of the total placental *Igf2 *expression.

By day 18 of pregnancy, when prepartum placental metabolic activity lessens, P0-*Igf2 *null pups have reduced placental and fetal weights [2, 5] and the magnitude of the upregulated placental glucose transport in this model falls [5]. We hypothesised that at this stage of pregnancy the fetal growth restriction is related to the placenta being less able to influence maternal metabolism and therefore a lowering of maternal glucose concentrations. Our results are not consistent with this, however, despite there being the expected fall in fasting insulin sensitivity as parturition approached. Perhaps in this case a fetal genotype effect on maternal glucose concentrations is just not required given that the affected placentas are already more efficient at nutrient transport [18]. Whilst in the current model, apart from a slight rise in maternal glucose concentrations 180 minutes after the glucose injection on day 16 of pregnancy which would have not been significant if correction was made for multiple testing, we could find no evidence of raised maternal glucose concentrations in pregnant mice carrying affected pups; affected female pups from these pregnancies are probably themselves at increased risk of having higher glucose concentrations in their own pregnancies given links between fetal growth restriction and the subsequent risk of gestational diabetes in humans [19] and raised glucose concentrations in rodent pregnancies [20].

## 5. Conclusion

In conclusion, the previously observed increased rates of placental glucose transport in fetuses that are P0-*Igf2 *null [5] and relative preservation of fetal body weight at day 16 of pregnancy are not related to increased maternal glucose concentrations. In fact, rather than stimulate it, fetal genotype effects on maternal metabolism appear to be diminished in this model by ablating P0-*Igf2 *transcription.

## Figures and Tables

**Table 1:** Blood glucose concentrations across an intra-peritoneal glucose tolerance test in control and experimental mice on day 1 of pregnancy. Data are presented as mean (SD), in mmol/L unless stated otherwise.

Time (min.)	Experimental group (*n* = 10)	Control group (*n* = 10)	*P*
0	6.3 (1.0)	6.6 (1.0)	.7
15	14.9 (3.7)	14.4 (1.9)	.7
30	14.3 (4.0)	12.3 (1.4)	.4
60	10.4 (4.3)	8.6 (0.8)	.1
120	6.9 (1.1)	6.4 (0.6)	.5
180	6.3 (0.8)	6.0 (0.7)	.8

Glucose area under the curve (mmol·min/L)	1662 (395)	1495 (113)	.3
